# Correlation analysis of serum IGF-1 and IL-6 and urinary albumin/creatinine ratio in patients with type 2 diabetic kidney disease

**DOI:** 10.3389/fendo.2022.1082492

**Published:** 2022-12-09

**Authors:** Guan-Wen Liu, Jiao-E Zeng, Lan-Fang Li

**Affiliations:** ^1^ Department of Endocrinology, Jingzhou Hospital Affiliated to Yangtze University, Jingzhou, Hubei, China; ^2^ Department of Endocrinology, Jingzhou Cental Hospital, Jingzhou, China

**Keywords:** insulin-like growth factor-1, IL-6, diabetic kidney disease, diabetes mellitus, urinary albumin/creatinine ratio

## Abstract

**Objectives:**

Diabetic kidney disease (DKD) is one of the most common chronic complications in diabetic patients, and there are major limitations in its pathological diagnosis. This study’s objectives were to examine the changes in serum insulin-like growth factor-1 (IGF-1) and interleukin-6 (IL-6) levels in DKD patients with various urinary albumin/creatinine ratio (ACR) and to evaluate the utility of these two biological markers in the clinical diagnosis of the condition.

**Methods:**

We chose 80 type 2 diabetic patients as the experimental group and 20 healthy normal participants as the control group. The experimental group was split into three groups based on the ACR range: diabetes without nephropathy group (ACR < 30 mg/g), microalbuminuric group (30 < ACR < 300 mg/g), and macroalbuminuric group (ACR > 300 mg/g). The levels of serum IL-6 and IGF-1 were assessed in each trial participant.

**Results:**

Serum IGF-1 was higher in the experimental group than in the control group (*P* < 0.01), and serum IL-6 levels were also higher than in the control group (*P* < 0.001). In DKD patients, serum levels of IL-6 and IGF-1 tended to rise when ACR levels rose. By Pearson correlation analysis, serum IGF-1 and IL-6 were positively correlated with ACR (r = 0.765 and r = 0.651, all *P* < 0.001) and negatively correlated with eGFR (r = -0.389 and r = -0.364, all *P* < 0.01). Additionally, the receiver operating characteristic (ROC) characteristic curve showed that the area under the curve (AUC) values for serum IGF-1 and IL-6 were 0.9056 and 0.7850, respectively, while the AUR value for both combined was 0.9367.

**Conclusion:**

Serum IGF-1 and IL-6 levels can be used to diagnose DKD, and the combined analysis of these two indicators can improve the sensitivity and specificity of the disease diagnosis.

## Introduction

Currently, chronic complications of diabetes mellitus are in serious form worldwide. According to reports, 30–40% of diabetic patients have DKD (1), which can eventually develop end-stage renal disease (ESRD) and is one of the leading causes of death in diabetic patients. Moreover, diabetic kidney disease accounts for 30%-50% of all patients with chronic renal failure requiring hemodialysis (2). The typical pathological changes in DKD include glomerular basement membrane thickening, glomerulosclerosis, tubulointerstitial fibrosis, interstitial inflammatory infiltration and podocyte injury (3). Patients in the early stages do not have overt clinical symptoms. The clinical symptoms of early DKD patients are not obvious, and the disease has commonly progressed to the middle and advanced stages by the time the diagnosis is made. Studies have shown that prognosis of patients with DKD can be improved by aggressive interventions (4, 5). Therefore, early diagnosis and timely treatment are crucial for patients with DKD. In recent years, the role of serum IGF-1 and serum IL-6 in the development of DKD has received considerable attention from scholars.

IGF-1 is a peptide substance with a function and structure particularly similar to insulin (6). IGF-1 plays an essential role in the regulation of cell development and growth and in increasing substance metabolism and other physiological processes. Early studies have shown that elevated serum levels of IGF-1 can influence the early pathological processes of diabetic kidney disease, such as glomerular enlargement, renal hyperfiltration and renal hypertrophy (7). In addition, IGF-1R inhibitors reduced tubulointerstitial fibrosis and renal inflammatory cell infiltration in DKD mice (8).

IL-6 is a member of the chemokine family that is commonly produced by fibroblasts and T Lymphocytes. Its primary biological role is to modulate the immune response and inflammation responses (9). It was found that the expression level of IL-6 mRNA was increased in mesangial cells and tubular cells of DKD patients (10). IL-6 can be closely associated with specific miRNAs, renal podocyte injury and involved in the pathogenesis of DKD (11). Additionally, IL-6 can also directly promote the production of extracellular matrix and proliferation of mesenchymal cells, thereby promoting the development of renal fibrosis. Our study investigated the diagnostic significance of each biological index and the combination of both in DKD by looking at serum IGF-1 and serum IL-6 levels in DKD patients with different ACR.

## Materials and methods

Eighty cases of type 2 diabetic patients (the diagnostic criteria of diabetes mellitus referred to the diagnostic criteria of diabetes mellitus published by WHO expert committee in 1999) who attended the inpatient department of endocrinology in Jingzhou Central Hospital from September 2021 to February 2022 were selected as the experimental group, and 20 cases of normal people who visited the health check-up center of the hospital during the same period were selected as the control group. According to the Mogensen staging and Kidney Disease Improving Global Outcomes (KDIGO) guidelines, the experimental group was divided into three groups: diabetes without nephropathy group (20 cases, ACR < 30 mg/g), microalbuminuric group (30 cases, 30 mg/g < ACR < 300 mg/g) and macroalbuminuric group (30 cases, ACR > 300 mg/g). The exclusion criteria for the DKD were as follows: patients with kidney disease caused by other diseases; patients who may affect the ACR level after treatment with drugs such as ACEI or ARB; factors that may affect IGF-1 level, such as tumors, tuberculosis, surgery, trauma, etc., are excluded, factors that may cause the increase in ACR were excluded, such as strenuous exercise in a short period of time, infection, and chronic heart failure.

## Experimental methods

### Measurement of general indicators

The sex, age and duration of diabetes mellitus were counted in all study subjects, height and weight were measured using a uniform tool, and body mass index (BMI) was calculated according to the BMI formula (BMI = weight/height ^2^, kg/m^2^). After 8h fast, blood samples were taken from patients in the morning and brought to our lab in less than two hours. Serum creatinine (SCr), total cholesterol (TC), IL-6, triglycerides (TG), albumin (ALB), and total protein (TP) levels from serum were measured using an automated biochemical analyzer. Fasting plasma glucose (FPG) measured by glucose oxidation method. Calculation of estimated glomerular filtration rat (eGFR) was performed using the modified Chinese MDRD formula. Serum IGF-1 was detected by chemiluminescence immuno-sandwich assay and detected by a MAGLUI series chemiluminescence analyzer produced by Shenzhen New Industry Biomedical Engineering Co., LTD. In addition, ACR from morning urine was detected by immunoturbidimetric assay.

### Statistical treatment

SPSS22.0 statistical software was used for statistical analysis of the data. For comparisons between two groups, all measures were expressed as mean ± SD when they conformed to normal distribution and variance homogeneity, and t-test was used to compare differences between groups; otherwise, the data were analyzed using the nonparametric Mann-Whitney test. For comparison of multiple data groups, one-way ANOVA and multiple comparisons were used, and for data that did not obey a normal distribution, the nonparametric Kruskal-Wallis test was used for analysis. Correlation analysis was performed using Pearson’s method or non-parametric Spearman. A *p* < 0.05 was considered statistically significant.

## Results

### Clinical indices of the healthy control group and the experimental group.

**Figure 1:**
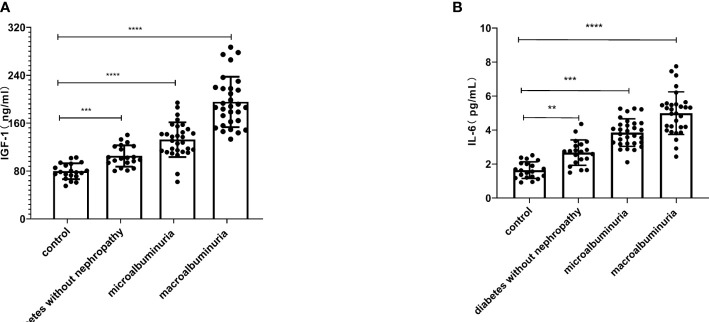
Serum insulin-like growth factor-1 (IGF-1) **(A)** and interleukin-6 (IL-6) **(B)** were higher in the diabetes without nephropathy, microalbuminuria and macroalbuminuria groups than in the control group (**P < 0.01, ***P < 0.001, ****P < 0.0001).

The general data of all experimental and control groups in **Table 1** were analyzed, and there was no statistical significance for age, gender, BMI, TG, TC, TP and ALB in the four groups (*P* > 0.05). Diabetes without nephropathy group, microalbuminuria group and macroalbuminuria group had higher levels of HbA1c and FPG than the healthy control group (*P* < 0.01, **Table 1**). In particular, we found that serum IGF-1 and IL-6 levels were significantly lower in the control group than in the diabetes without nephropathy group, the macroalbuminuria group and the microalbuminuria group (*P* < 0.05) (**Figure 1**).

**Table 1:** Clinical data comparison between the control group and the experimental group.

Group	Control	Diabetes without nephropathy	Microalbuminuria	Macroalbuminuria
Number	20	20	30	30
Age (years)mean +/- SE	57.45 ± 12.38	59.10 ± 8.53	57.60 ± 10.45	59.53 ± 10.22
Gender (female/male)	11/9	12/8	18/12	22/8
Course of disease (years) (interquartile)	–	6.0 (1-15)	9.0 (1-26)^a^	9.0 (1-31)^a^
BMI (kg/m2)mean +/- SE	24.63 ± 2.50	26.54 ± 3.60	25.15 ± 3.32	24.60 ± 3.22
eGFR (ml/min)mean +/- SE	–	107.87 ± 27.98	86.66 ± 28.40^d^	67.70 ± 24.51^df^
TP (g/L)mean +/- SE	69.90 ± 4.55	69.89 ± 4.55	71.08 ± 6.24	70.06 ± 6.13
ALB (g/L)mean +/- SE	41.76 ± 3.80	42.19 ± 3.21	43.73 ± 3.37	42.59 ± 4.46
TC (mmol/L)mean +/- SE	4.34 ± 0.94	4.63 ± 1.20	4.46 ± 1.05	4.63 ± 0.88
TG (mmol/L)mean +/- SE	1.52 ± 0.56	1.54 ± 0.58	1.72 ± 0.74	1.74 ± 0.84
FPG (mmol/L)mean +/- SE	5.25 ± 0.41	8.49 ± 1.25^b^	8.01 ± 1.56^b^	7.76 ± 1.49^b^
IL-6 (pg/mL)mean +/- SE	1.64 ± 0.49	2.68 ± 0.75^b^	3.85 ± 0.81^bd^	5.00 ± 1.25^bdf^
IGF-1 (ng/ml)	79.65 ± 13.23	105.21 ± 17.80^b^	132.48 ± 29.03^bd^	195.49 ± 42.25^bdf^
ACR (mg/g)	–	9.28 ± 4.72	148.94 ± 71.13^d^	556.66 ± 197.51^df^
HbA1c (%)	5.20 ± 0.27	7.62 ± 1.60^b^	8.10 ± 1.67^b^	8.30 ± 1.96^b^
DR (yes/no)	–	3/17	30/30^d^	30/30^d^

Data are means ± SD for Gaussian variables and median (interquartile) for non-Gaussian variables.

Diabetes without nephropathy, Microalbuminuric and Macroalbuminuric versus Control, ^a^ *P* < 0.05, ^b^ *P* < 0.01; Macroalbuminuric and Microalbuminuric versus diabetes without nephropathy, ^c^ *P* < 0.05, ^d^ *P* < 0.01; Microalbuminuric versus Macroalbuminuric, ^e^ *P* < 0.05, ^f^ *P* < 0.01.

BMI, body mass index; DR, diabetic retinopath; eGFR, estimated glomerular filtration rat; TP, total protein; ALB, albumin; TC, total cholesterol; FPG, fasting plasma glucose; TG, triacylglycerol; IL-6, interleukin 6; IGF-1, insulin-like growth factor-1; HbA1c, glycated hemoglobin; ACR, urinary albumin/creatinine ratio.

### Serum IGF-1 and IL-6 levels in diabetic patients with different ACR.

**Figure 2:**
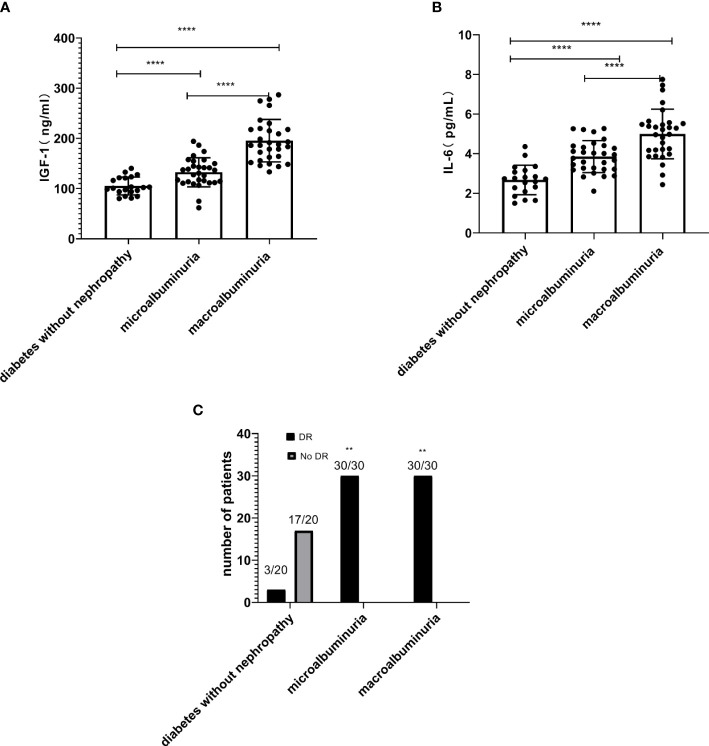
The levels of insulin-like growth factor-1 (IGF-1) **(A)** and interleukin-6 (IL-6) **(B)** increased with the increase of urinary albumin/creatinine ratio (ACR) in diabetic kidney disease (DKD) patients (****P < 0.0001). **(C)** The number of DKD patients with DR is higher than the diabetes without nephropathy group (**P < 0.01).

In this study, serum IGF-1 and IL-6 levels were significantly higher in the macroalbuminuria group (195.49 ± 42.25, 5.00 ± 1.25, *P* < 0.0001, **Table 1**, **Figure 2**) than in the microalbumin group (132.48 ± 29.03, 3.85 ± 0.81, *P* < 0.0001, **Table 1**, **Figure 2**). Moreover, both groups were higher than the group with diabetes without nephropathy (105.21 ± 17.80, 2.68 ± 0.75, *P* < 0.0001, **Table 1**, **Figure 2**). This indicates that serum IGF-1 and IL-6 levels increased with the increase of ACR in DKD patients. And significantly more patients in the microalbuminuria and macroalbuminuria groups had diabetic retinopathy (DR) than diabetic patients without nephropathy (**Figure 2**).

### Correlation analysis of serum IGF-1, serum IL-6 and other clinical indicators in DKD patients

**Figure 3:**
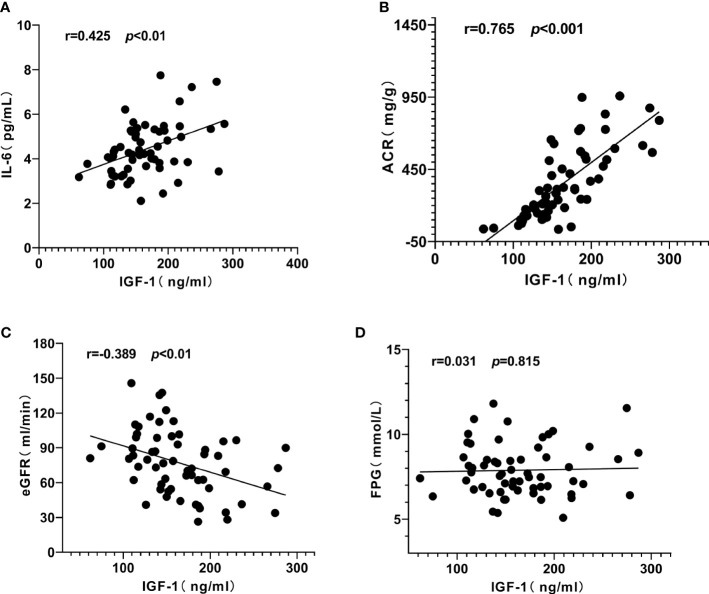
Correlation analysis of serum insulin-like growth factor-1 (IGF-1) with interleukin-6 (IL-6), urinary albumin/creatinine ratio (ACR) and estimated glomerular filtration rat (eGFR) in patients with diabetic kidney disease (DKD). **(A)** IGF-1 was positively correlated with IL-6. **(B)** IGF-1 was positively correlated with ACR. **(C)** IGF-1 was negatively correlated with eGFR. **(D)** IGF-1 had no significant correlation with fasting plasma glucose (FPG).

Pearson correlation analysis was used to explore the close degree of serum IGF-1 and clinical indicators in patients with diabetic kidney disease. The results showed that serum IGF-1 was positively correlated with serum IL-6 (r = 0.425, *P* < 0.01) (**Figure 3A**) and ACR (r = 0.765, *P* < 0.001) (**Figure 3B**) to varying degrees and negatively correlated with eGFR (r = - 0.389, *P* < 0.01) (**Figure 3C**). There was no significant correlation with duration of diabetes, albumin, total protein and fasting blood glucose (*P* > 0.05) (**Table 1**, **Figure 3D**).

**Figure 4:**
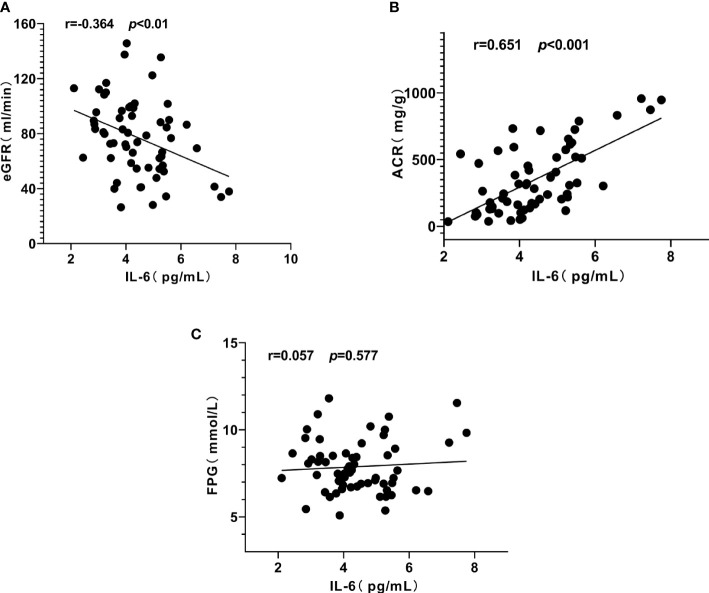
Correlation between interleukin-6 (IL-6), urinary albumin/creatinine ratio (ACR) and estimated glomerular filtration rat (eGFR) in patients with diabetic kidney disease (DKD). **(A)** IL-6 was negatively correlated with eGFR. **(B)** IL-6 was positively correlated with ACR. **(C)** IL-6 had no significant correlation with fasting plasma glucose (FPG).

Similarly, serum IL-6 was positively correlated with ACR (r = 0.651, *P* < 0.001) (**Figure 4B**), IGF-1 (r = 0.425, *P* < 0.01) (**Figure 3A**) and negatively correlated with eGFR (r = - 0.364, *P* < 0.01) (**Figure 4A**), but not significantly correlated with FPG (*P* = 0.577, **Figure 4C**).

### Logistic regression analysis

Logistic regression analysis was performed to correlate the progression of DKD with serum IL-6, serum IGF-1, TG, TC, FPG and HbA1c levels. The results showed that the progression of DKD was correlated with serum IGF-1 and IL-6 (*P* < 0.05, **Table 2**), independent of other indicators (*P >* 0.05, **Table 2**).

**Table 2:** Logistic regression analysis of clinical biological indicators and the progression of DKD.

	B	SE	Wald	Sig.	Exp(B)	95%Cl	
IL-6	2.230	0.879	6.438	0.011	9.304	1.661	52.109
IGF-1	0.089	0.030	8.609	0.003	1.093	1.030	1.160
FPG	-0.552	0.672	0.676	0.411	0.576	0.154	2.147
TC	0.619	0.772	0.643	0.423	1.858	0.140	4.801
TG	-0.199	0.902	0.049	0.825	0.819	0.140	4.801
HbA1c	0.344	0.505	0.463	0.496	1.410	0.524	3.794

### ROC curves for the separation and combination of serum IGF-1 and serum IL-6

**Figure 5:**
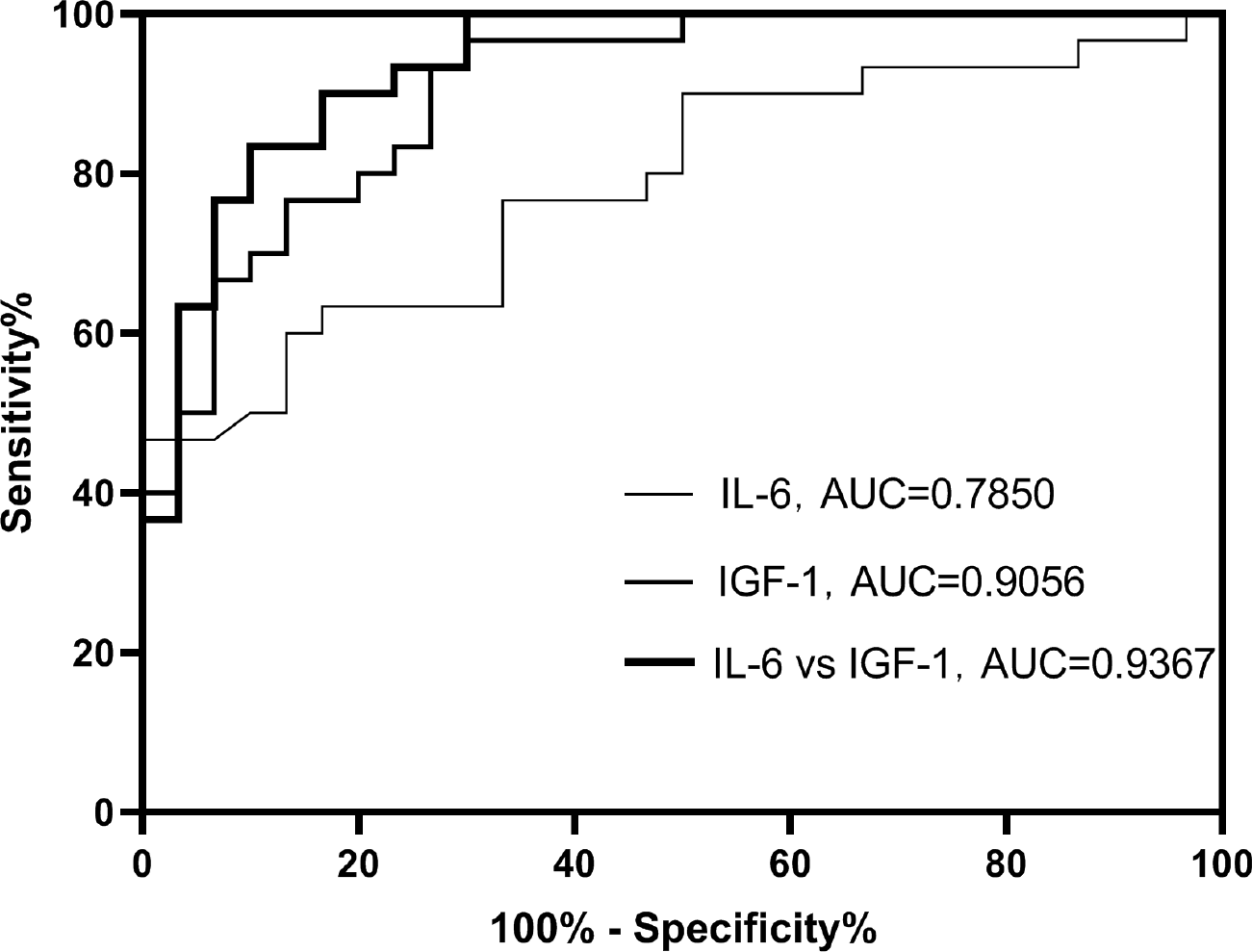
ROC curves for the separation and combination of serum IGF-1 and serum IL-6.

On the basis of examination of logistic regression analysis, we additionally established ROC curves. In this study, the area under the ROC curve was used to indicate the accuracy of biological indicators to predict disease. As shown, AUC values for serum IGF-1 and IL-6 were 0.9056 and 0.7850 (**Figure 5**), respectively, and AUC value for both combined was 0. 9367 (**Figure 5**).

Moreover, the sensitivity and specificity of the serum IGF-1 index were 96.7% and 70.0% (**Table 3**), respectively, and the sensitivity and specificity of serum IL-6 were 66.3% and 83.0% (**Table 3**), respectively. The sensitivity and specificity of the combination of the two indicators were 90.0% and 83.3% (**Table 3**), respectively. This indicates not only that serum IGF-1 and IL-6 can be used to predict DKD progression separately, but also that the combination of the two has higher sensitivity and specificity.

**Table 3:** Analysis of ROC curve results.

	AUC	p-Value	95%Cl	Specificity, %	Sensitivity, %
IGF-1	0.9056	<0.0001	(0.833, 0.978)	70.0	96.7
IL-6	0.7850	<0.0001	(0.669, 0.901)	83.0	66.3
combined	0.9367	<0.0001	(0.878, 0.995)	83.3	90.0

## Discussion

DKD is one of the most serious chronic consequences in people with diabetes and is the second most deadly condition for people with type 2 diabetes after cardiovascular disease. Histopathologic diagnoses are the gold standard for the diagnosis of DKD, but this test is a traumatic examination with limitations and low patient willingness to be tested. Therefore, it poses difficulties for clinical diagnosis. ACR is frequently used to assist in the diagnosis and staging of DKD, however it can be interfered with by other factors. Previous studies have demonstrated an association between serum IGF-1 and the development of DKD (7). This is consistent with the results of our study, where serum IGF-1 levels were higher in the group of patients with DKD than in the control group. Moreover, serum IGF-1 levels continued to rise as the ACR index increased. As an insulin-like analogue, IGF-1 has a role in promoting cell proliferation, differentiation and accelerating substance metabolism (12). Previous reports have shown that the expression of IGF-1, IGF-1 mRNA and IGFBP1 mRNA was upregulated in the kidneys of rats with DKD compared with normal controls (13, 14). Levin et al. showed that increased IGF-1 levels were associated with early pathological changes in STZ-induced diabetic rats, including renal hypertrophy and hyperfiltration (7). In addition, elevated levels of IGF-1 activate the Akt signaling pathway, which expresses Snail1 and results in renal fibrosis (8, 15). Kong et al. showed that the activation of IGF-1/IGF-1R pathway can promote renal mesangial cells to produce type IV collagen and connexin, which are the main components of extracellular matrix and can promote the proliferation of mesangial cells and stromal thickening, thus leading to glomerular sclerosis (16). In addition, elevated IGF-1 expression levels were associated with other renal histopathological alterations, including mesangial expansion, infiltration of inflammatory factors, and renal cell proliferation (17, 18). Our study also found that IL-6 expression level was significantly elevated in DKD patients and associated with ACR. Inflammatory response is one of the pathogenic mechanisms of DKD (19). Studies have shown that gene polymorphisms of IL-6 are closely related to the development of DKD and its elevated expression level can increase the probability of the disease (20). IL-6 can not only directly stimulate mesangial cells proliferation, damage islet cells and disrupt islet function, but also damage renal vascular endothelial cells and promote renal interstitial fibrosis. IL-6 receptor blockers have been reported to inhibit the inflammatory response and reduce insulin resistance in DKD mice (21). In addition, by inhibiting the IL-6R/JAK2/STAT3 pathway in renal cells was able to protect the kidney in diabetic rats (22).

In this study, we confirmed the promoting effect of serum IGF-1 and serum IL-6 expression levels on DKD, based on which logistic regression analysis was performed and ROC curves were constructed. The results showed that serum IGF-1 and IL-6 contributed to the diagnosis of DKD, with AUR values of 0.9056 and 0.7850 (**Table 3**), respectively. Moreover, the sensitivity and specificity of the two indicators combined to diagnose the disease was higher, with an AUR value of 0.9367 (**Table 3**). The majority of the IGF-1 in the organism exists in bound form. IGF-1 binding to IGF-1R activates the downstream MAPK pathway, increases the expression of fibrosis-related genes and stromal Has2 genes, and accelerates the process of mesangial expansion and proteinuria in streptozotocin-induced diabetic mice (23). Through oxidative stress and inflammatory reactions, IL-6 may contribute to DKD (24, 25). Studies have shown that by inhibiting the p38-MAPK signaling pathway, the level of the inflammatory factor IL-6 can be attenuated, thereby reducing renal inflammatory infiltration (26). Both serum IGF-1 and IL-6 are involved in the development of DKD, and we speculate that this may be related to the coordinated action of both signaling pathways, which together promote the progression of DKD.

Correlation analysis showed that serum IGF-1 and IL-6 were negatively correlated with eGFR and positively correlated with ACR. It is hypothesized that serum IGF-1 and IL-6 exacerbate renal injury by affecting glomerular filtration rate as well as urinary albumin. Additionally, we discovered that the number of individuals with DR was greatly lower in the diabetes without nephropathy group than in the microalbuminuric and macroalbuminuric groups (**Figure 2C**). We hypothesize that this may be because serum IGF-1 and IL-6 are involved in the development of retinopathy. Increased IGF-1 levels were reported to hasten the onset of DR (27). IL-6 was found to be positively linked with the development of DR. Inhibiting the activation of the IL-6/STAT3 signaling pathway and altering oxidative stress and inflammatory response allowed IL-6 to play a protective role in the retina of diabetic rats (28). The relationship between serum IGF-1 and IL-6 expression levels and the severity of DR is unclear, and more studies are feasible for us to follow.

In conclusion, serum IGF-1 and IL-6 can be used as biochemical indicators for diagnosing and judging the progression of DKD, which is closely related to the kidney damage of the disease, and the combination of the two can improve the sensitivity and specificity of the assay, which has guiding significance for the subsequent diagnosis and treatment of DKD. However, there are still some limitations in this study, and the sample size can be expanded in the future to further explore the changes of serum IGF-1 and serum IL-6 levels in DKD of different severity.

## Data availability statement

The original contributions presented in the study are included in the article/**Supplementary Material**. Further inquiries can be directed to the corresponding author.

## Ethics statement

The studies involving human participants were reviewed and approved by Jingzhou Cental Hospital. The patients/participants provided their written informed consent to participate in this study.

## Author contributions

The study was designed by J-EZ, and data extraction and analysis were collected by G-WL. The initial manuscript was written by G-WL and finally revised by L-FL and J-EZ. All authors contributed to the article and approved the submitted version.

## References

[1] Umanath K Lewis JB. Update on diabetic nephropathy: Core curriculum 2018. Am J Kidney Dis (2018) 71(6):884–95. doi: 10.1053/j.ajkd.2017.10.026 29398179

[2] Ruiz-Ortega M Rodrigues-Diez RR Lavoz C Rayego-Mateos S. Special issue ‘Diabetic nephropathy: Diagnosis, prevention and treatment’. J Clin Med (2020) 9(3):813. doi: 10.3390/jcm9030813 32192024 PMC7141346

[3] Vartak T Godson C Brennan E. Therapeutic potential of pro-resolving mediators in diabetic kidney disease. Advanced Drug Delivery Rev (2021) 178:113965. doi: 10.1016/j.addr.2021.113965 34508793

[4] Andrésdóttir G Jensen ML Carstensen B Parving H-H Hovind P Hansen TW. Improved prognosis of diabetic nephropathy in type 1 diabetes. Kidney Int (2015) 87(2):417–26. doi: 10.1038/ki.2014.206 24918158

[5] Sofie Astrup A Tarnow L Rossing P Pietraszek L Riis Hansen P Parving H-H. Improved prognosis in type 1 diabetic patients with nephropathy: A prospective follow-up study. Kidney Int (2005) 68(3):1250–7. doi: 10.1111/j.1523-1755.2005.00521.x 16105058

[6] Shi G-J Shi G-R Zhou J Zhang W Gao C Jiang Y. Involvement of growth factors in diabetes mellitus and its complications: A general review. Biomed Pharmacother (2018) 101:510–27. doi: 10.1016/j.biopha.2018.02.105 29505922

[7] Levin-Iaina N Iaina A Raz I. The emerging role of NO and IGF-1 in early renal hypertrophy in STZ-induced diabetic rats. Diabetes/Metabolism Res Rev (2011) 27(3):235–43. doi: 10.1002/dmrr.1172 21309053

[8] Dong R Yu J Yu F Yang S Qian Q Zha Y. IGF-1/IGF-1R blockade ameliorates diabetic kidney disease through normalizing Snail1 expression in a mouse model. Am J Physiology-Endocrinol Metab (2019) 317(4):E686–98. doi: 10.1152/ajpendo.00071.2019 31361542

[9] Chang AS Hathaway CK Smithies O Kakoki M. Transforming growth factor-β1 and diabetic nephropathy. Am J Physiol - Renal Physiol (2016) 310(8):F689–96. doi: 10.1152/ajprenal.00502.2015 PMC4835922 26719364

[10] Suzuki D Miyazaki M Naka R Koji T Yagame M Jinde K. *In situ* hybridization of interleukin 6 in diabetic nephropathy. Diabetes (1995) 44(10):1233–8. doi: 10.2337/diab.44.10.1233 7556963

[11] Alina-Emanuela G Gadalean F Vlad A Vlad M Victor D Vlad D. MO635PRO-inflammatory cytokines IL-6 and IL-17 display a particular molecular pattern in association with dysregulated mirnas in patients with type 2 diabetes mellitus in the early stages of diabetic kidney disease. Nephrol Dialysis Transplant (2021) 36(Supplement_1). doi: 10.1093/ndt/gfab094.003

[12] Park J Yan G Kwon K-C Liu M Gonnella PA Yang S. Oral delivery of novel human IGF-1 bioencapsulated in lettuce cells promotes musculoskeletal cell proliferation, differentiation and diabetic fracture healing. Biomaterials (2020) 233:119591. doi: 10.1016/j.biomaterials.2019.119591 31870566 PMC6990632

[13] Bach LA Dean R Youssef S Cooper ME. Aminoguanidine ameliorates changes in the IGF system in experimental diabetic nephropathy. Nephrol Dialysis Transplant (2000) 15(3):347–54. doi: 10.1093/ndt/15.3.347 10692520

[14] Du XY Zheng BT Pang Y Zhang W Liu M Xu XL Zhou SJ. The potential mechanism of INHBC and CSF1R in diabetic nephropathy. Eur Rev Med Pharmacol Sci (2020) 24(4):1970–8. doi: 10.26355/eurrev_202002_20374 32141565

[15] Wang W Sun W Cheng Y Xu Z Cai L. Role of sirtuin-1 in diabetic nephropathy. J Mol Med (2019) 97(3):291–309. doi: 10.1007/s00109-019-01743-7 30707256 PMC6394539

[16] Kong Y Shen Y Ni J Shao D Miao N Xu J. Insulin deficiency induces rat renal mesangial cell dysfunction *via* activation of IGF-1/IGF-1R pathway. Acta Pharmacologica Sin (2016) 37(2):217–27. doi: 10.1038/aps.2015.128 PMC4753370 26775660

[17] Gurevich E Segev Y Landau D. Growth hormone and IGF1 actions in kidney development and function. Cells (2021) 10(12):3371. doi: 10.3390/cells10123371 34943879 PMC8699155

[18] Nambam B Schatz D. Growth hormone and insulin-like growth factor-I axis in type 1 diabetes. Growth Hormone IGF Res (2018) 38:49–52. doi: 10.1016/j.ghir.2017.12.005 29249623

[19] Pérez-Morales RE Del Pino MD Valdivielso JM Ortiz A Mora-Fernández C Navarro-González JF. Inflammation in diabetic kidney disease. Nephron (2018) 143(1):12–6. doi: 10.1159/000493278 30273931

[20] Chen B Wu M Zang C Li Y Xu Z. Association between IL-6 polymorphisms and diabetic nephropathy risk: A meta-analysis. Am J Med Sci (2019) 358(5):363–73. doi: 10.1016/j.amjms.2019.07.011 31451183

[21] Wu R Liu X Yin J Wu H Cai X Wang N. IL-6 receptor blockade ameliorates diabetic nephropathy *via* inhibiting inflammasome in mice. Metabolism (2018) 83:18–24. doi: 10.1016/j.metabol.2018.01.002 29336982

[22] Zhang N Zheng Q Wang Y Lin J Wang H Liu R. Renoprotective effect of the recombinant anti-IL-6R fusion proteins by inhibiting JAK2/STAT3 signaling pathway in diabetic nephropathy. Front Pharmacol (2021) 12:681424. doi: 10.3389/fphar.2021.681424 34054555 PMC8155588

[23] Ishikado A Shinjo T Yokomizo H Maeda Y Park K Qi W. 309-or: IGF-1 receptors, not insulin receptors, on mesangial cells are accelerating mesangial expansion and albuminuria in streptozotocin-induced diabetic mice. Diabetes (2020) 69(Supplement_1). doi: 10.2337/db20-309-or

[24] Cui J Zhang X Guo C Zhang L. The association of interieukin-6 polymorphism (rs1800795) with microvascular complications in type 2 diabetes mellitus. Bioscience Rep (2020) 40(10). doi: 10.1042/bsr20201105 PMC7569201 33016995

[25] Lou Z Li Q Wang C Li Y. The effects of microrna-126 reduced inflammation and apoptosis of diabetic nephropathy through PI3K/akt signalling pathway by VEGF. Arch Physiol Biochem (2020) 128(5):1265–74. doi: 10.1080/13813455.2020.1767146 32449863

[26] Li J Bao L Zha D Zhang L Gao P Zhang J Wu X. Oridonin protects against the inflammatory response in diabetic nephropathy by inhibiting the TLR4/p38-MAPK and TLR4/NF-KB signaling pathways. Int Immunopharmacol (2018) 55:9–19. doi: 10.1016/j.intimp.2017.11.040 29207360

[27] Liu X Li J Li X. Mir-142-5p regulates the progression of diabetic retinopathy by targeting IGF1. Int J Immunopathol Pharmacol (2020) 34:205873842090904. doi: 10.1177/2058738420909041 PMC7052454 32116075

[28] Wang Y Zhai WL Yang YW. Association between NDRG2/IL-6/STAT3 signaling pathway and diabetic retinopathy in rats. Eur Rev Med Pharmacol Sci (2020) 24(7):3476–84. doi: 10.26355/eurrev_202004_20806 32329820

